# Deprivation, clubs and drugs: results of a UK regional population-based cross-sectional study of weight management strategies

**DOI:** 10.1186/1471-2458-14-444

**Published:** 2014-05-12

**Authors:** Clare Relton, Jessica Li, Mark Strong, Michelle Holdsworth, Richard Cooper, Mark Green, Paul Bissell

**Affiliations:** 1 Public Health Section, ScHARR, University of Sheffield, Regent Court, 30 Regent Street, Sheffield S1 4DA, UK

**Keywords:** Obesity, Weight management strategy, Population, Survey, Cohort, Slimming clubs, Weight loss medication

## Abstract

**Background:**

Despite rising levels of obesity in England, little is known about slimming club and weight loss drug (medication) use or users. In order to inform future commissioning, we report the prevalence of various weight management strategies and examine the associations between slimming club and medication use and age, gender, deprivation and body mass index.

**Methods:**

A population based cross-sectional survey of 26,113 adults was conducted in South Yorkshire using a self-completed health questionnaire. Participants were asked whether they had ever used the following interventions to manage their weight: increasing exercise, healthy eating, controlling portion size, slimming club, over the counter weight loss medication, or meal replacements. Factors associated with slimming club and weight-loss medication use were explored using logistic regression.

**Results:**

Over half of the sample was either overweight (36.6%) or obese (19.6%). Obesity was more common in the most deprived areas compared to the least deprived (26.3% vs. 12.0%). Healthy eating (49.0%), controlling portion size (43.4%), and increasing exercise (43.0%) were the most commonly reported weight management strategies. Less common strategies were attending a slimming club (17.2%), meal replacements (3.4%) and weight-loss medication (3.2%). Adjusting for BMI, age, deprivation and long standing health conditions, women were significantly more likely to report ever using a slimming club (adjusted OR = 18.63, 95% CI = 16.52–21.00) and more likely to report ever using over the counter weight-loss medications (AOR = 3.73, 95% CI = 3.10-4.48), while respondents from the most deprived areas were less likely to report using slimming clubs (AOR = 0.60, 95% CI = 0.53-0.68), and more likely to reporting using weight loss medications (AOR =1.38, 95% CI = 1.05-1.82).

**Conclusion:**

A large proportion of individuals report having used weight management strategies. Slimming clubs and over-the-counter weight loss medication account for a smaller proportion of the overall uptake. Those from less deprived areas were more likely to use slimming clubs while those from more deprived areas were more likely to use weight-loss medications. Future NHS and Local Authority commissioning of weight management services must be aware of this varying social gradient in weight management strategies.

## Background

Rising levels of obesity and obesity related conditions in England pose a major health problem which requires the attention of policy makers, health professionals and the public alike [1]. The majority of men and women in the United Kingdom (UK) are concerned about their weight [2] and use a variety of strategies to help manage their weight including: increasing exercise, healthy eating, controlling portion size, slimming club, prescription and over the counter weight loss medication, and meal replacements. When this study was conceived the numbers of weight management services being offered in the community NHS was increasing in line with the multi-component approach advocated by NICE Obesity Guidance [3]. However, little was known about the prevalence of slimming clubs and weight loss drug (medication) use or the characteristics of users. In order to help inform the design of future local National Health Service (NHS) and Local Authority policy and commissioning of weight management services, up to date information on the prevalence of slimming clubs and weight loss medications and the factors associated with their usage is required.

### Slimming clubs

In the UK, a number of high profile internationally recognised commercial slimming clubs (e.g. Weight Watchers, Slimming World) provide support for people who wish to manage their weight, and in some areas of the UK, NHS clinicians refer overweight and obese patients to NHS funded slimming clubs and weight management programmes. The majority of people attending both NHS and commercial slimming clubs are obese (rather than overweight), middle-aged and female [4,5]. The most recent population based survey was conducted over a decade ago (2002) and collected information on slimming club use, using a stratified probability sample of British adults; 18.1% of women and 1.6% of men sampled reported using a slimming club in the previous 3 years [2].

### Weight loss medications

A range of medicines have been used in the management of obesity. Traditionally these have been amphetamine-related appetite suppressants, but the withdrawal of sibutramine in Europe [6] and the cannabinoid-related rimonabant (due to increased cardiovascular risk) marked the end of amphetamines marketed for weight-loss on prescription. Orlistat is now the only licensed weight-loss medication in the UK for obesity and is available via prescription on the NHS (as Xenical) and over-the counter through pharmacies at a lower strength (as Alli).Though the contra-indications (malabsorption) and side effects (fecal incontinence and frequent or urgent bowel movements) of orlistat are known, little is known about the demographics of orlistat users. Population-based estimates of usage rates for weight loss drugs, as well as information on the types of users, are scant. One UK study of adults attending dietetic clinics found that men were less likely to use special slimming products than women (OR = 0.30) [7]. Another UK study found that 2.0% of women and 0.2% of men used ‘pills or injections’ to lose weight [2], while 14% of adults reported using over the counter appetite suppressants, herbal products or weight-loss supplements in a US study [7]. UK and US studies [7,8] both report that those who had made more attempts at losing weight were more likely to use weight loss medications or dietary supplements. Use of dietary supplements has also been found to be associated with being female, obese, younger, less educated, lower income, in poorer health, and having a greater concern about one’s weight [8].

### Study aims

To report the prevalence of a number of weight management strategies used by the South Yorkshire adult population and to examine the associations between slimming club and medication use and a range of explanatory factors (age, gender, deprivation and body mass index (BMI)).

## Methods

### Design, setting, and recruitment

We conducted a population based cross-sectional survey of 27,806 adults (aged 16 – 85 yr) using a self-completed health questionnaire. Participants were recruited to the South Yorkshire Cohort [9] using a two stage sampling method between June 2010 and December 2012. The first stage involved recruiting General Practices (GPs) to take part in the study (n = 43). In the second stage, patients aged 16-85 registered with these practices were invited to complete postal or online health questionnaires. The health questionnaire (see Additional file 1) collected the following information: gender, date of birth, number of children, self-reported height, weight and waist measurement (tape measure was provided), ethnicity, life satisfaction, health-related quality of life using EuroQoL-5D, [10] long-standing illness, health-problem, condition or disability (for example diabetes, cancer, stroke), educational qualifications obtained, and socio-economic status using the National Statistics Socio-economic Classification self-coded version. Patients were asked if they had a long standing illness, health problem, condition or disability (with 12 categories provided). Information on physical activity levels and weight management strategies were also collected. NHS Research Ethics Committee approval for the South Yorkshire Cohort protocol was obtained on 27th April, 2010 (REC ref: 09/H1306/97).

### Weight management measures

Patients were asked “Is managing your weight a concern for you?” and whether they had ever used the following interventions to help manage their weight: increasing exercise, healthy eating, controlling portion size, using a slimming club (Slimming World, Weightwatchers, Rosemary Conley Diet and Fitness Club, Lighterlife), using over the counter weight loss medication, and using meal replacements. We defined the category ‘ever used a slimming club’ and included in this respondents who reported having ever used Slimming World, Weight Watchers, or Rosemary Conley Diet and Fitness Club. As Lighterlife members do not meet with other Lighterlife ‘club’ members we excluded Lighterlife from our list of slimming clubs for the purposes of our analysis (n = 128).

Patients were also asked whether they had ever used over the counter weight loss medications, and two options given: ‘Alli (orlistat)’, ‘Others (please describe)’. The residential postcode for each respondent was linked to the corresponding Lower Super Output Area and hence to the area’s 2010 Indices of Deprivation (ID) score. Respondents’ ID 2010 scores were then categorised by national quintile. The variable was useful for measuring social inequalities in weight management strategy, as the index captures the multi-dimensional aspects of neighbourhood deprivation (e.g. income, health, crime etc). Age was recoded into four categories: 16-35, 36-55, 56-75, 76+. BMI, based on self-reported weight and height, was categorised as normal or underweight (BMI less than 25), overweight (BMI greater than or equal to 25 and less than 30), and obese (BMI greater than or equal to 30). As there were just 428 respondents (1.64%) who were classified as underweight, underweight was categorized with normal.

### Statistical analyses

Population data from the 2011 Census for South Yorkshire were used to create sampling weights that adjusted for the under sampling of men, younger individuals, and more deprived areas. The reweighted sample (with respect to gender, age, and deprivation to reflect the South Yorkshire population) was used to estimate proportions for those using slimming clubs and weight-loss medications in the South Yorkshire adult population.

Chi-square tests were used to determine significant factors to include in the analysis. Multivariate logistic regression models were used to estimate odds ratios (OR) for risk factors across two outcome variables: having ever attended a slimming club and having ever used over the counter weight-loss medications. Models adjusted for age, gender, deprivation quintile, and BMI. As we anticipated that having a long-standing illness health problem, condition or disability would be a confounder, we adjusted for this in an additional model. All statistical analyses were conducted using STATA version 12.0.

## Results

156,866 questionnaires were sent out and 27,806 were returned (response rate = 15.9%). Records with missing data on age (n = 409), BMI (n = 1408), and ID quintile (n = 75) were excluded from the analysis leaving a total sample size of 26,113.

### Sample characteristics

The sample characteristics are shown in Table 1. The mean age of respondents was 55 (range 16-86, SD = 17.08). Over half of the sample was either overweight (36.6%) or obese (19.6%). BMI significantly increased with level of deprivation (χ^2^ (df = 8, n = 26113) = 537.4; p < 0.001); the proportion of obese people within the most deprived quintile was double that in the least deprived quintile (26.3% compared to 12.0%). More than half of the sample (61.4%) reported having one or more long-standing illness, health problem, condition, or disability.

**Table 1:** Sample characteristics for the South Yorkshire Cohort (N = 26113)

		**Gender**		**Age (y)**				**Deprivation**				
	**Overall**	**Male (n = 11460)**	**Female (n = 14653)**	**16-35 (n = 4344)**	**36-55 (n = 7962)**	**56-75 (n = 11212)**	**76+ (n = 2595)**	**Least deprived (n = 3795)**	**Low deprivation (n = 6612)**	**Average (n = 4348)**	**High deprivation (n = 4823)**	**Most deprived (n = 6535)**
**Height (cm)**	169.0 (10.0)	176.7 (7.5)	162.9 (7.1)	170.1 (10.2)	170.0 (9.9)	168.4 (9.9)	166.4 (9.9)	170.1 (10.0)	169.4 (9.9)	169.2 (9.9)	168.5 (10.0)	168.0 (10.2)
**Weight (kg)**	75.7 (16.5)	83.7 (15.0)	69.5 (14.8)	71.3 (16.7)	77.0 (17.3)	77.2 (16.0)	72.8 (13.7)	73.5 (14.9)	74.4 (15.4)	75.8 (16.2)	76.7 (16.6)	77.5 (18.2)
**Waist (cm)**	89.9 (13.5)	94.7 (11.5)	86.1 (13.7)	83.7 (13.3)	88.9 (13.2)	92.2 (13.0)	93.3 (12.7)	87.4 (12.3)	88.5 (12.5)	89.7 (13.2)	90.7 (13.4)	92.5 (14.8)
**BMI (%)**												
**Normal/underweight**	43.8	37.2	48.9	63.9	44.1	36.0	42.4	52.6	48.2	43.5	38.9	37.9
**Overweight**	36.6	43.7	31.1	23.3	36.2	41.2	40.7	35.4	36.6	37.4	38.0	35.8
**Obese**	19.6	19.1	20.1	12.8	19.8	22.8	16.9	12.0	15.2	19.1	23.1	26.3
Health condition (%)*												
**No**	38.6	36.8	40.0	67.6	50.6	24.9	12.1	46.1	41.6	39.7	36.2	32.1
**Yes**	61.4	63.2	60.1	32.4	49.5	75.1	87.9	54.0	58.4	60.3	63.8	67.9

Figures are in the format mean (s.d) unless otherwise stated.

*Any long-standing illness, health problem, condition or disability.

### Concern with weight management

Table 2 reports the proportion of the sample who are concerned with weight and the proportion who engage in various weight management strategies by age, gender and deprivation. Despite a greater proportion of men being overweight or obese in the study sample, more women reported concern about managing their weight (45.3%) compared to men (30.8%). Concern with managing weight was highest amongst those aged 36-55 and lowest amongst those over 75, but there were no statistically significant differences in weight concern across deprivation groups.

**Table 2:** Percentage of people using weight-loss management strategies within the South Yorkshire Cohort: column % (N = 26113)

		**Gender**		**Age**				**Deprivation**				
	**Overall**	**Male (n = 11460)**	**Female (n = 14653)**	**16-35 (n = 4344)**	**36-55 (n = 7962)**	**56-75 (n = 11212)**	**76+ (n = 2595)**	**Least deprived (n = 3795)**	**Low deprivation (n = 6612)**	**Average (n = 4348)**	**High deprivation (n = 4823)**	**Most deprived (n = 6535)**
**Weight concern**												
No	57.1	65.3	50.8	55.6	51.1	58.3	73.1	57.9	58.7	56.0	56.5	56.3
Yes	38.9	30.8	45.3	40.6	45.4	37.8	21.0	39.4	38.0	40.0	38.9	38.9
Missing	4.0	4.0	4.0	3.8	3.5	4.0	5.9	2.7	3.4	4.0	4.5	4.9
**Increasing exercise**												
No	57.0	62.5	52.7	41.6	43.8	65.3	87.4	49.6	53.4	54.3	58.2	65.9
Yes	43.0	37.5	47.3	58.4	56.2	34.7	12.6	50.4	46.6	45.7	41.8	34.1
**Healthy eating**												
No	51.0	59.5	44.5	50.3	45.4	52.1	64.9	46.5	48.6	48.2	51.5	57.7
Yes	49.0	40.5	55.5	49.7	54.6	47.9	35.1	53.5	51.5	51.8	48.5	42.3
**Controlling portion size**												
No	56.6	64.6	50.3	59.4	53.8	55.4	65.9	54.6	54.8	56.0	56.2	60.2
Yes	43.4	35.4	49.7	40.7	46.2	44.6	34.1	45.4	45.2	44.0	43.8	39.8
**Ever used slimming club**												
No	82.8	97.2	71.6	83.3	78.4	83.5	92.8	83.6	82.5	80.8	81.6	85.0
Yes	17.2	2.8	28.4	16.7	21.6	16.5	7.2	16.4	17.5	19.2	18.5	15.0
**Ever used weight-loss medication**												
No	96.8	98.7	95.4	94.2	96.1	97.9	99.1	98.1	97.6	96.9	96.3	95.7
Yes	3.2	1.3	4.6	5.8	3.9	2.1	0.9	1.9	2.4	3.1	3.8	4.3
**Ever used meal replacement**												
No	96.7	98.1	95.5	95.6	95.9	97.2	98.2	97.2	97.4	96.6	96.0	96.1
Yes	3.4	1.9	4.5	4.4	4.1	2.8	1.8	2.9	2.6	3.5	4.0	3.9

### Strategies for managing weight

The most commonly reported weight management strategies (ever used) in the sample were healthy eating (49.0%), controlling portion size (43.4%), and increasing exercise (43.0%) (Table 2). Using slimming clubs, weight-loss medications, or meal replacements were the least commonly reported weight management strategies out of the options provided. Women were significantly more likely than men to report ever using any of the listed weight-management strategies.

Increasing exercise was the most popular strategy used by those aged 16-35 and 36-55. Healthy eating, controlling portion size, and having ever used a slimming club were most commonly reported by those aged 36-55 and least commonly reported by those aged 76+. A wide variety of different types of exercise were reported in the free text sections (e.g. walking, karate, football, aerobics class, Zumba class). Many slimming clubs were NHS funded (e.g. RIO [11]), although others were commercial or were provided free in the workplace or by groups of friends. Respondents also reported using a number of different meal replacements, e.g. The Cambridge Diet, Herbalife, SlimFast and Lipotrim.

### Slimming clubs

Less than a fifth (17.2%) of the sample reported having ever attended a slimming club (Table 2). After weighting the sample for age, sex, and deprivation, the estimated population proportion is 15.8% (95% CI = 15.3% to 16.3%). Slimming club attenders were significantly more likely to be female (p < 0.001); 28.4% of women sampled had used a slimming club compared to only 2.8% of men. Those aged 36-55 were more likely to have ever attended a slimming club (21.6%) followed by those aged 16-35 (16.7%) and 56-75 (16.5%). Only 7.2% of older adults aged 76+ had ever attended a slimming club. Slimming club use was lowest in the most deprived quintile (15.0%) (Table 2).

Table 3 shows specific slimming clubs and weight-loss medications by gender, age, deprivation, and BMI. The most frequently used slimming clubs were WeightWatchers (estimated population proportion 10.7% [95% CI = 10.3 to 11.1]) and Slimming World (estimated population proportion 8.5% [95% CI = 8.2 to 8.9). Less than 2% of the sample had attended the Rosemary Conley Diet and Fitness Club.

**Table 3:** Percentage of those who ever attended a slimming club or used weight-loss medication by gender, age, deprivation, and BMI: row % (N = 26113)

	**Overall**	**Overall weighted (95% CI)**	**Gender**		**Age**				**Deprivation**					**BMI**		
			**Male**	**Female**	**16-35**	**36-55**	**56-75**	**76+**	**D1**	**D2**	**D3**	**D4**	**D5**	**BMI 1**	**BMI 2**	**BMI 3**
**Slimming world** (n = 2376)	9.1	8.5 (8.2-8.9)	6.5	93.5	16.2	40.0	40.8	3.0	12.1	26.0	18.3	20.3	23.3	19.2	38.5	42.3
**Weightwatchers** (n = 3063)	11.7	10.7 (10.3-11.1)	6.4	93.6	16.2	38.4	40.8	4.6	15.2	25.0	18.6	19.5	21.7	21.4	40.6	38.0
**Rosemary Conley Diet and Fitness Club** (n = 449)	1.7	1.4 (1.3-1.6)	5.4	94.7	6.7	44.3	45.4	3.6	16.0	28.7	22.1	19.4	13.8	20.0	44.3	35.6
**Lighterlife** (n = 127)	0.5	0.5 (0.4-0.6)	17.3	82.7	16.5	49.6	25.2	8.7	12.6	21.3	19.7	20.5	26.0	15.0	31.5	53.5
**Alli (orlistat)** (n = 561)	2.2	2.3 (2.1-2.5)	18.7	81.3	24.6	39.2	33.3	2.9	8.4	19.8	17.1	21.0	33.7	7.8	26.0	66.1
**Other medication** (n = 336)	1.3	1.6 (1.4-1.8)	17.3	82.7	38.7	34.8	21.7	4.8	9.2	16.7	15.8	22.3	36.0	28.3	35.1	36.6

DI = Least Deprived; D2 = Low deprivation; D3 = Average; D4 = High deprivation; D5 = Most deprived.

BMI 1 = Normal/underweight; BMI 2 = Overweight; BMI 3 = Obese.

As mentioned previously, Lighterlife combines the promotion of meal replacements with group meetings and one-to-one counselling, but members do not meet each other as they do in a conventional slimming club, therefore a separate analysis was conducted to compare Lighterlife users to conventional slimming club users. Compared to the slimming clubs listed, a higher proportion of Lighterlife users were men (17.3% compared to only 6.5% of those using Slimming World [χ^2^ (1, n = 2503) = 21.7; p < 0.001], 6.4% of those using Weightwatchers [χ^2^ (1, n = 3190) = 23.1; p < 0.001], and 5.4% of those using Rosemary Conley Diet and Fitness Club [χ^2^ (1, n = 576) = 19.3; p < 0.001]). With regards to age, Lighterlife had the highest proportion of older users, i.e. aged 76+ (8.7%) and Rosemary Conley Diet and Fitness Club had the lowest proportion of younger users, i.e. aged 16-35 (6.7%) compared to other clubs. A higher proportion of respondents from the most deprived groups had also used Lighterlife and those who reported using Lighterlife were more likely to be obese than users of Slimming World, Weightwatchers, and Rosemary Conley Diet and Fitness Club.

### Factors associated with attending slimming clubs

Table 4 presents factors associated with having ever used a slimming club. The likelihood of ever attending a slimming club was substantially higher for females, adults aged 36-55 compared to adults aged 16-35, those from areas of average and high deprivation compared to the least deprived areas, and those who were overweight or obese compared to normal/underweight. The likelihood was lower for those aged 76+ compared to 16-35.When adjusting for these factors in the multivariate model (having a long-standing health condition was not found to be significantly associated with having ever attended a slimming club and was therefore excluded from the multivariate model to improve the model fit), the adjusted odds remained significantly higher for females, those aged 36-55 compared to 16-35, and those who were overweight or obese while the adjusted odds were significantly lower for those aged 56+ and those from high or the most deprived areas (Table 4).

**Table 4:** Factors associated with ever using slimming clubs: n, % unadjusted and multivariate logistic regression

	**Never used slimming clubs (n = 21628) n (%)**	**Used slimming clubs (n = 4485) n (%)**	**Unadjusted OR**	**95% CI**	**p value**	**Adjusted OR**	**95% CI**	**p value**
**Gender**								
Male	11136 (51.5)	324 (7.2)	1			1		
Female	10492 (48.5)	4161 (92.8)	13.63	(12.14-15.31)	<0.001	18.63	(16.52-21.00)	<0.001
**Age**								
16-35	3617 (16.7)	727 (16.2)	1			1		
36-55	6244 (28.9)	1718 (38.3)	1.37	(1.24-1.51)	<0.001	1.29	(1.16-1.44)	<0.001
56-75	9360 (43.3)	1852 (41.3)	0.98	(0.90-1.08)	0.743	0.77	(0.70-0.86)	<0.001
76+	2407 (11.1)	188 (4.2)	0.39	(0.33-0.46)	<0.001	0.28	(0.23-0.34)	<0.001
**Deprivation**								
Least deprived	3174 (14.7)	621 (13.9)	1			1		
Low deprivation	5455 (25.2)	1157 (25.8)	1.08	(0.97-1.21)	0.139	1.00	(0.89-1.13)	0.949
Average	3514 (16.3)	834 (18.6)	1.21	(1.08-1.36)	0.001	1.05	(0.92-1.20)	0.431
High deprivation	3933 (18.2)	890 (19.8)	1.16	(1.03-1.29)	0.011	0.87	(0.77-1.00)	0.040
Most deprived	5552 (25.7)	983 (21.9)	0.90	(0.81-1.01)	0.074	0.60	(0.53-0.68)	<0.001
**BMI**								
Normal/underweight	10416 (48.2)	1008 (22.5)	1			1		
Overweight	7765 (35.9)	1796 (40.0)	2.39	(2.20-2.60)	<0.001	4.09	(3.73-4.48)	<0.001
Obese	3447 (15.9)	1681 (37.5)	5.04	(4.62-5.50)	<0.001	8.63	(7.80-9.54)	<0.001
**Long-standing illness, health problem, condition or disability**								
No	8133 (38.6)	1684 (38.3)	1					
Yes	12934 (61.4)	2708 (61.7)	1.01	(0.95-1.08)	0.745			

OR = Odds-ratio; CI = Confidence interval.

### Weight-loss medications

Within our sample, 3.2% of participants reported having ever used weight-loss medication (Table 2). The estimated population proportion, adjusted for sampling strategy, is 3.7% (95% CI = 3.5% to 3.9%). Of those who reported using over the counter weight-loss medications, 562 (68.0%) reported using orlistat. Other commonly self-reported weight-loss medications were herbal medications and herbal supplements (e.g. Acai, Adios, Green tea capsules, Thermabole, Seakelp and Zotrim), amphetamine-related appetite suppressants (such as sibutramine (Reductil®), phentramine (Iomin®), and Ponderax®) and laxatives. Thirty-five respondents reported using meal replacements including Celebrity Slim, Complan, Lipotrim, Ultra Slim, and various Slim Fast products.

Weight-loss medication use was highest amongst female and overweight or obese respondents (Table 3). Furthermore, weight-loss medication users were more likely to be young and from deprived areas (Tables 2 and 3). Orlistat use was highest amongst those aged 36-55, while other over the counter weight-loss medication use was highest amongst younger adults (aged 16 – 35) and over half of those who reported ever using orlistat or other medications were from high or the most deprived areas.

### Factors associated with using weight-loss medications

Table 5 lists those factors associated with ever using weight-loss medications. The unadjusted odds ratios shows that the likelihood of having ever used weight-loss medications was higher for females, those from average/more deprived areas, those who were overweight or obese, and those with a long-standing illness, health problem, condition or disability. The odds of having ever used weight-loss medications was lower for those aged over 35. After adjusting for these factors in the multivariate model as well as having a long standing illness, health problem, condition or disability (as it was found to be associated with both BMI [χ^2^ (df = 2, n = 25455) = 976.02; p < 0.001] and having ever used a weight loss medication [χ^2^ (df = 2, n = 25455) = 22.64; p < 0.001]), the odds of having ever used over the counter weight-loss medication remained significantly higher for females, those from high or the most deprived areas compared to the least deprived areas, those who were overweight or obese compared to normal/underweight, and those with a long-standing illness, health problem, condition or disability. The odds remained lower for those aged 36 and over compared to those aged 16-35 (Table 5). There was also a significant interaction between being overweight and having a long-standing illness, health problem, condition or disability, suggesting that the relationship between BMI and using weight-loss medications is different for those who have or do not have a long-standing illness, health problem, condition or disability, and vice versa. For instance, in those who are normal (or under) weight, people with long standing illness have higher odds of using weight loss medication while being overweight reduces the strength of the effect of long standing illness on medication use.

**Table 5:** Factors associated with ever using weight-loss medications: n, % unadjusted and multivariate logistic regression

	**Never used weight-loss medications (n = 25287) n (%)**	**Used weight-loss medications (n = 826) n (%)**	**Unadjusted OR**	**95% CI**	**p value**	**Adjusted OR**	**95% CI**	**p value**
**Gender**								
Male	11312 (44.7)	148 (17.9)	1			1		
Female	13975 (55.3)	678 (82.1)	3.71	(3.10-4.44)	<0.001	3.73	(3.10-4.48)	<0.001
**Age**								
16-35	4091 (16.2)	253 (30.6)	1			1		
36-55	7651 (30.3)	311 (37.7)	0.66	(0.55-0.78)	<0.001	0.47	(0.39-0.57)	<0.001
56-75	10973 (43.4)	239 (28.9)	0.35	(0.29-0.42)	<0.001	0.20	(0.17-0.25)	<0.001
76+	2572 (10.2)	23 (2.8)	0.14	(0.09-0.22)	<0.001	0.09	(0.06-0.14)	<0.001
**Deprivation**								
Least deprived	3723 (14.7)	72 (8.7)	1			1		
Low deprivation	6455 (25.5)	157 (19.0)	1.26	(0.95-1.67)	0.111	1.16	(0.87-1.55)	0.317
Average	4213 (16.7)	135 (16.3)	1.66	(1.24-2.21)	0.001	1.34	(1.00-1.81)	0.053
High deprivation	4642 (18.4)	181 (21.9)	2.02	(1.53-2.66)	<0.001	1.34	(1.00-1.79)	0.047
Most deprived	6254 (24.7)	281 (34.0)	2.32	(1.79-3.02)	<0.001	1.38	(1.05-1.82)	0.021
**BMI**								
Normal/underweight	11300 (44.7)	124 (15.0)	1			1		
Overweight	9318 (36.9)	243 (29.4)	2.38	(1.91-2.96)	<0.001	4.87	(3.42-6.92)	<0.001
Obese	4669 (18.5)	459 (55.6)	8.96	(7.33-10.96)	<0.001	13.34	(9.34-19.05)	<0.001
Health condition*								
No	9569 (38.8)	248 (30.6)	1			1		
Yes	15079 (61.2)	563 (69.4)	1.44	(1.24-1.68)	<0.001	2.34	(1.61-3.38)	<0.001
**BMI x Health condition**								
Overweight x Yes						0.56	(0.36-0.89)	0.013
Obese x Yes						0.72	(0.46-1.11)	0.138

OR = Odds-ratios; CI = confidence interval.

*Any long-standing illness, health problem, condition or disability.

## Discussion

This study investigated the self-reported weight management strategies of the South Yorkshire adult population and examined the characteristics of those using slimming clubs and weight loss medications. Adults in this UK region report using a wide variety of weight management strategies. Use of different weight management strategies varied by level of deprivation. Almost half of our sample report attempting to manage their weight using practices consistent with public health messages [12]: healthy eating (49%), increasing exercise (43%), and controlling portion size (43%). Slimming club (17%), weight-loss medication (3%), and meal replacements use (3%), remain at similar levels to those reported a decade ago [2].

A traditional social gradient was displayed for increasing exercise, controlling portion size and healthy eating, with these practices being more common in the least deprived areas. We found that slimming club and medication use differs by deprivation; those from less deprived areas were more likely to use slimming clubs while those from more deprived areas were more likely to use weight-loss medications. There was an inverse social gradient for the use of meal replacements and over the counter weight loss medication, showing the prominence of difference practices by deprivation (albeit with much lower values).

### Limitations

The generalisability of the study results from this one region to other UK regions is unknown. The response to the survey was low (15.9%), although no incentives were offered for completion of the survey. The low response rate limits the representativeness of the results, particularly for men, younger individuals, black and minority ethnics and those living in more deprived areas (although we adjusted for gender, age, and deprivation through sampling weights). There may also be a saliency bias if responders were more concerned about their health than non-responders. Self-reported data may be limited by the memory of respondents. In order to keep our model parsimonious and focus on deprivation and BMI, we did not control for other potential confounders such as smoking, pain, and mobility, though we feel that adjusting for long-standing health illnesses, health problems, conditions or disabilities could have partially accounted for these. The long-term health variable used did not discriminate by condition and therefore is a limitation of our analysis. Our list of closed questions limited the range of weight management strategies reported in this study.

### Changing nature of slimming clubs

While studying our results, the question as to what constituted a slimming club arose. We had imagined that all slimming clubs included the following components: regular face to face meetings with expert led group discussion, weekly weighing/measuring and group face to face disclosure. However, responses revealed a number of changes. Firstly, slimming clubs are now frequently described as ‘Weight management programmes’ (particularly those provided/funded by the NHS). Secondly, that other components are included, e.g. meal replacements, weight loss medications, and one-to-one counselling. Thirdly, many slimming clubs/weight management programmes are now delivered using online and mobile phone technologies, Personal Digital Assistants in addition to the more traditional methods (face to face, books and magazines). For example, WeightWatchers offers both WeightWatcher meetings and/or WeightWatchers online. Other slimming clubs provide online chat rooms, motivational DVDs and videos, text reminders, and weight management apps which track food, weight and activity. Future research on slimming clubs should take account of the new terminologies, non-traditional additional components and multiple channels of delivery now being used by slimming clubs.

### Interpretation

Over a quarter (28.4%) of women sampled had used a slimming club compared to 2.8% of men (ratio of 9:1), which is lower than the 12:1 ratio reported a decade ago [2]. Women were also much more likely to report concern about their weight compared to men, despite more men being overweight or obese in the study sample.

There were differences in the demographics with regards to age, gender, and deprivation between Lighterlife and all the other slimming clubs. Lighterlife combines a very low calorie diet regime (VLCDs) with one-to-one counselling. The VLCD may attract those who wish to achieve rapid weight loss and the one to one counselling may be attractive to men, who may feel more uncomfortable than women attending group meetings integral to Slimming World and Weight Watchers [13].

Those in the most deprived quintile were least likely to report managing their weight by increasing exercise, healthy eating, controlling portion size or using a slimming club. However, those in the most deprived quintile were more likely to report use of weight loss-medications or meal replacements. Future local NHS and Local Authority health and public health commissioning must take into consideration this varying social gradient in weight management strategies when deciding on the optimum method of allocating and targeting resources to improve the weight management strategies used in their area.

## Conclusion

A large proportion of individuals report having used weight management strategies. Slimming clubs and over-the-counter weight loss medication account for a smaller proportion of the overall uptake.

Those from less deprived areas were more likely to use slimming clubs while those from more deprived areas were more likely to use weight-loss medications. Future NHS and Local Authority commissioning of weight management services must be aware of this varying social gradient in deciding how to optimise resources to improve the weight management strategies used their region.

## Competing interests

The authors declare that they have no competing interests.

## Authors’ contributions

CR conceived the idea for the article, JL led and conducted the data analysis. JL, CR and MS wrote and commented on all drafts. MG, PB, RC and MH commented on later drafts. All authors read and approved the final manuscript.

## Pre-publication history

The pre-publication history for this paper can be accessed here:

http://www.biomedcentral.com/1471-2458/14/444/prepub

## Supplementary Material

Additional file 1 Pdf of Health Questionnaire. Click here for file
