# Supplementary material for: Deprivation, clubs and drugs: results of a UK regional population-based cross-sectional study of weight management strategies
Source: BMC Public Health. 2014 May 12;14:444. doi: 10.1186/1471-2458-14-444 (PMC4046155; doi:10.1186/1471-2458-14-444)
Supplement: Additional file 1 — Pdf of Health Questionnaire. [file 1471-2458-14-444-S1.pdf]

Consent Form

Thank you for your answers. These will be combined with thousands of others. What we learn will help us understand how we can improve the health of people living in South Yorkshire.

May we contact you again?

☐ Yes ☐ No

May we use the information you provide to look at the benefit of health treatments?

☐ Yes ☐ No

May we look at your health records?

☐ Yes ☐ No

Please fill in your **forename**, **surname** and **address** if different to those printed to the left:

Forename (print)

Surname (print)

Address

Postcode

Please fill in your preferred contact details below:

Telephone

Mobile phone*

Email*

*Optional

Signature . . . .

Thank you for completing the Health Questionnaire. Please put it in the prepaid envelope and post it to the researchers. NO STAMP NEEDED.

If you have any queries or require further information about this study please contact Dr Clare Relton or Dr Joanna Blackburn at ScHARR, University of Sheffield, FREEPOST - SF1314, Sheffield, S1 1AY.

Tel: 0114 222 0796 **Email:** syc@sheffield.ac.uk **Online questionnaire:** http://syc.shef.ac.uk

© NIHR CLAHRC for South Yorkshire

NHS

Health Questionnaire

South Yorkshire Cohort

Welcome to the South Yorkshire Cohort! Please help us by filling in this questionnaire. Your answers will help us understand how we can improve the health of people living in South Yorkshire.

The questionnaire will take 5-10 minutes to complete. When you have completed the questionnaire, please put it in the envelope and post it to the researchers at the University of Sheffield. You do not need a stamp.

You can also fill in the questionnaire on **Facebook** (South Yorkshire Cohort) or by going to: **http://syc.shef.ac.uk** and following the instructions.

Please return the questionnaire as soon as you can, ideally within 3 weeks.

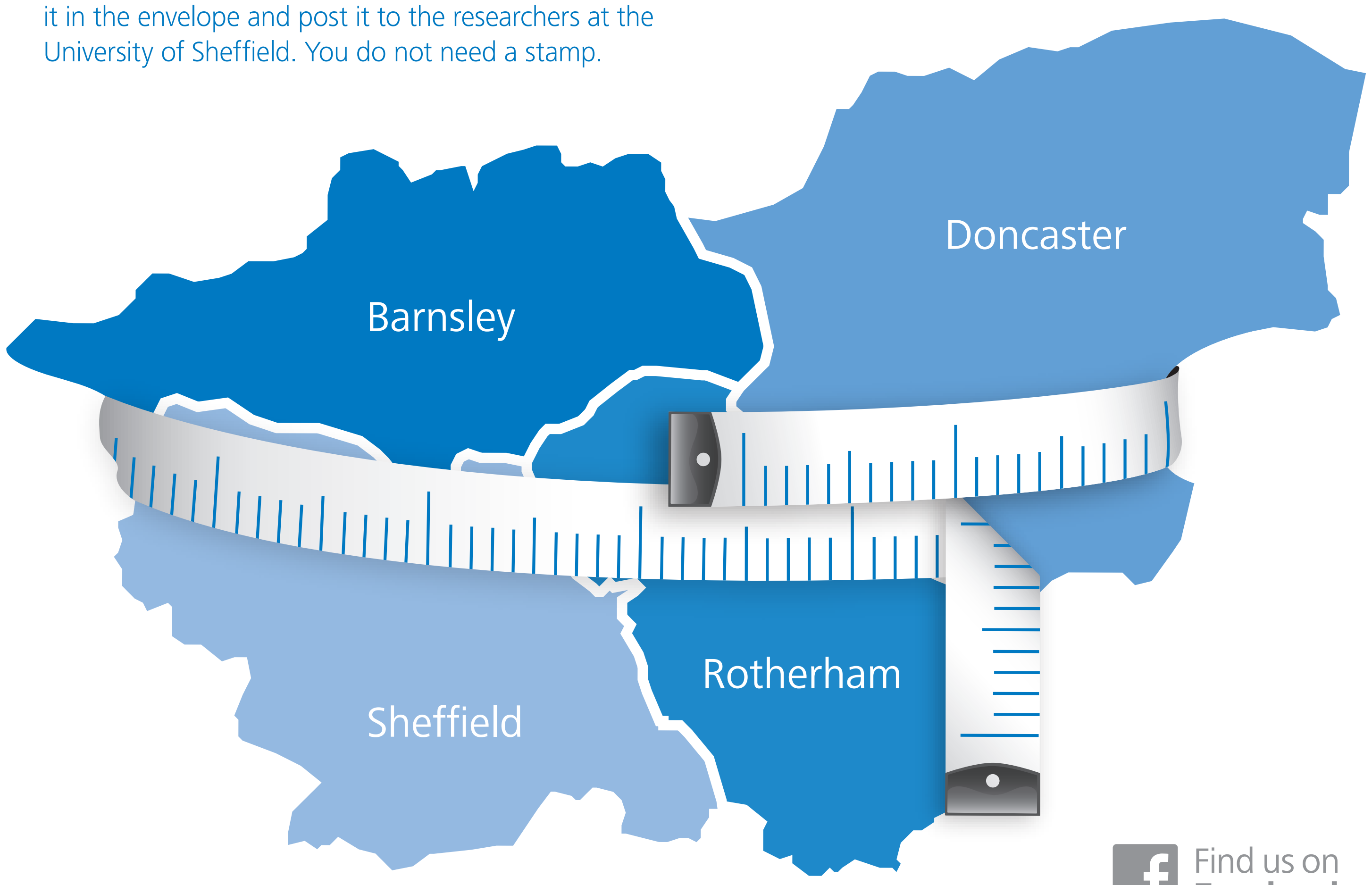

Find us on Facebook

NIHR CLAHRC for South Yorkshire

The University Of Sheffield.

Sheffield Hallam University

About you

Your sex ☐ Male ☐ Female

Your date of birth ☐☐ ☐☐ ☐☐☐☐
d d m m y y y y

How many children do you have (under 18)? ☐☐

Your height ☐ feet ☐☐ inches **OR** ☐☐☐ cm

Your weight ☐☐ stone ☐☐ lbs **OR** ☐☐☐ kgs

Your waist measurement ☐☐ inches **OR** ☐☐☐ cm

Please use the tape measure enclosed and measure at the narrowest point between your hips and your ribs, usually just above the belly button.

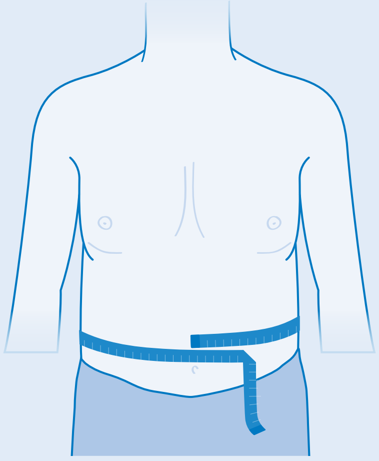

Which ethnic group do you belong to?

White

☐ British
☐ Irish
☐ Other white background

Asian or Asian British

☐ Indian
☐ Pakistani
☐ Bangladeshi
☐ Other Asian background

Mixed

☐ White and Black Caribbean
☐ White and Black African
☐ White and Asian
☐ Other mixed background

Black or Black British

☐ Caribbean
☐ African
☐ Other Black background

Chinese or other ethnic group

☐ Chinese
☐ Other ethnic group

Gypsy / traveller

☐ Gypsy / traveller

Thinking about your own life and personal circumstances, how satisfied are you with your life as a whole?

Completely Dissatisfied										Completely Satisfied
0	1	2	3	4	5	6	7	8	9	10
☐	☐	☐	☐	☐	☐	☐	☐	☐	☐	☐

You and your work

Are you currently employed? ☐ Yes ☐ No

The following questions refer to your current main job, or (if you are not working now) to your last main job. Please tick one box only per question.

Employee or self-employed: Do (did) you work as an employee or are (were) you self-employed?
☐ Employee ☐ Self-employed with employees ☐ Self-employed / freelance without employees

Number of employees: *For employees:* indicate below how many people work (worked) for your employer at the place where you work (worked).
For self-employed: indicate below how many people you employ (employed).
☐ 1-24 ☐ 25 or more

Supervisory Status: Do (did) you supervise any other employees?
A supervisor or foreman is responsible for overseeing the work of other employees on a day-to-day basis
☐ Yes ☐ No

Occupation

Please tick one box to show which **best** describes the sort of work you do. *(If you are not working now, please tick a box to show what you did in your last job). PLEASE TICK **ONE BOX** ONLY*

☐ **Modern professional occupations** *such as:* teacher - nurse - physiotherapist - social worker - welfare officer - artist - musician - police officer (sergeant or above) - software designer

☐ **Clerical and intermediate occupations** *such as:* secretary - personal assistant - clerical worker - office clerk - call centre agent - nursing auxiliary - nursery nurse

☐ **Senior managers or administrators** (usually responsible for planning, organising and co-ordinating work and for finance) *such as:* finance manager - chief executive

☐ **Technical and craft occupations** *such as:* motor mechanic - fitter - inspector - plumber - printer - tool maker - electrician - gardener - train driver

☐ **Semi-routine manual and service occupations** *such as:* postal worker - machine operative - security guard - caretaker - farm worker - catering assistant - receptionist - sales assistant

☐ **Routine manual and service occupations** *such as:* HGV driver - van driver - cleaner - porter - packer - sewing machinist - messenger - labourer - waiter / waitress - bar staff

☐ **Middle or junior managers** *such as:* office manager - retail manager - bank manager - restaurant manager - warehouse manager - publican

☐ **Traditional professional occupations** *such as:* accountant - solicitor - medical practitioner - scientist - civil / mechanical engineer

During the last 3 MONTHS, how many days have you taken off from paid work as a result of ill health? ☐☐ days

During the last 3 MONTHS, on how many days has your ill health prevented you from carrying out your:
Household tasks: ☐☐ days
Leisure activities: ☐☐ days

Your health care

In the last 3 MONTHS, how many times have you visited the following:

Hospital	Times
Accident & Emergency (A&E)	☐☐
Hospital - day case	☐☐
Hospital - outpatients	☐☐
Hospital - in-patients (*how many nights*)	☐☐

Other healthcarers	Times
GP	☐☐
Nurse	☐☐
Physiotherapist	☐☐
Dietitian	☐☐
Midwife	☐☐
Mental health worker	☐☐
Psychotherapist	☐☐

Other carers	Times
Counsellor	☐☐
Care worker	☐☐
Social worker	☐☐
Health visitor	☐☐
Community health champion	☐☐
Health trainer	☐☐

Alternative therapist	Times
Acupuncturist	☐☐
Chiropractor	☐☐
Herbalist	☐☐
Homeopath	☐☐
Osteopath	☐☐

Other *Please describe:*	Times
	☐☐

You and your education

Which of these qualifications do you have?
Please tick all the qualifications that apply, or if not specified, the nearest equivalent:

1+ O Levels / CSE / GCSEs (any grades)	☐	NVQ Level 1, Foundation GNVQ	☐
5+ O Levels, 5+ CSEs (grade 1), 5+ GCSEs (grades A-C), School Certificate	☐	NVQ Level 2, Intermediate GNVQ	☐
1+ A Levels / AS Levels	☐	NVQ Level 3, Advanced GNVQ	☐
2+ A Levels, 4+ AS Levels, Higher School Certificate	☐	NVQ Levels 4-5, HNC, HND	☐
First Degree (e.g. BA, BSc)	☐	Other Qualifications (e.g. City & Guilds, RSA / OCR, BTEC / Edexcel)	☐
Higher Degree (e.g. MA, PhD, PGCE, Post-graduate Certificates / Diplomas)	☐	No qualifications	☐

Your health

Here are some simple questions about your health in general. By ticking one answer in each group below, please indicate which statements best describe your own health state TODAY.

**Mobility**	Please tick one:
I have no problems in walking about	☐
I have some problems in walking about	☐
I am confined to bed	☐
**Self-care**	Please tick one:
I have no problems with self-care	☐
I have some problems washing or dressing myself	☐
I am unable to wash or dress myself	☐
**Usual Activities**	Please tick one:
I have no problems with performing my usual activities (e.g. work, study, housework, family or leisure activities)	☐
I have some problems with performing my usual activities	☐
I am unable to perform my usual activities	☐
**Pain / Discomfort**	Please tick one:
I have no pain or discomfort	☐
I have moderate pain or discomfort	☐
I have extreme pain or discomfort	☐
**Anxiety / Depression**	Please tick one:
I am not anxious or depressed	☐
I am moderately anxious or depressed	☐
I am extremely anxious or depressed	☐

Long standing conditions

Do you have any long-standing illness, health problem, condition or disability? ☐ Yes ☐ No

If yes, please tick all that apply:

Tiredness / Fatigue	☐	High blood pressure	☐
Pain	☐	Heart disease	☐
Insomnia	☐	Osteoarthritis	☐
Anxiety / Nerves	☐	Stroke	☐
Depression	☐	Cancer	☐
Diabetes	☐	Other: *(please state)*	☐
Breathing problems e.g. chronic bronchitis, asthma or emphysema	☐		

Your health

Alcohol: How many days in the last WEEK did you drink alcohol? ☐ days

How many units of alcohol did you drink in the last WEEK? ☐☐ units

A unit of alcohol is equal to ½ a pint of ordinary beer, lager or cider, 1 single measure of spirits, 1 small glass of wine or 1 measure of fortified wine.

Smoking: Which of these best describes you?

☐ I smoke daily
☐ I smoke occasionally but not every day
☐ I used to smoke daily but now not at all
☐ I used to smoke occasionally but now not at all
☐ I have never smoked

Your medication

Are you currently taking any medication? (Either prescribed by your doctor, or that you buy yourself).

☐ Yes ☐ No

Please list all your medication below, including vitamins & mineral supplements, dietary supplements or diet pills, herbal or homeopathic remedies.

Name & strength of tablet, medicine, ointment, drops, inhaler or injection	Is this prescribed for you? *Please tick*:	What is this for?
(Example) Co-codamol 8mg/500mg tablets	☐ Yes ☑ No	*Joint pain*
	☐ Yes ☐ No	
	☐ Yes ☐ No	
	☐ Yes ☐ No	
	☐ Yes ☐ No	
	☐ Yes ☐ No	
	☐ Yes ☐ No	
	☐ Yes ☐ No	
	☐ Yes ☐ No	
	☐ Yes ☐ No	
	☐ Yes ☐ No	
	☐ Yes ☐ No	
	☐ Yes ☐ No	
	☐ Yes ☐ No	

Your exercise and food

During the last WEEK, how many hours did you spend on each of the following activities? *(Please tick)*

	None	Some but less than 1 hour	At least 1 hour but less than 3 hours	3 hours or more
Physical exercise such as swimming, jogging, aerobics, football, tennis, gym workout etc.	☐	☐	☐	☐
Cycling, including cycling to work and during leisure time	☐	☐	☐	☐
Walking, including walking to work, shopping, for pleasure etc.	☐	☐	☐	☐

Is managing your weight a concern for you? ☐ Yes ☐ No

Have you ever used any of the following to help you manage your weight? *(Please tick)*

Exercise and food	
Increasing your exercise	☐
Healthy eating *Please describe:*	☐
Controlling your portion size	☐
**Slimming Clubs**	
Slimming World	☐
Weightwatchers	☐
Rosemary Conley Diet and Fitness Club	☐
Lighterlife	☐
Other *Please describe:*	☐
**Over the counter weight loss medication**	
Alli (orlistat)	☐
Other *Please describe:*	☐
**Meal replacements**	
Lighterlife	☐
Other *Please describe:*	☐
